# Tick-Borne Encephalitis Virus Replication, Intracellular Trafficking, and Pathogenicity in Human Intestinal Caco-2 Cell Monolayers

**DOI:** 10.1371/journal.pone.0096957

**Published:** 2014-05-12

**Authors:** Chao Yu, Katharina Achazi, Lars Möller, Joerg D. Schulzke, Matthias Niedrig, Roland Bücker

**Affiliations:** 1 Centre for Biological Threats and Special Pathogens, ZBS 1: Highly Pathogenic Viruses, Robert Koch Institute, Berlin, Germany; 2 Institute of Chemistry and Biochemistry, Freie Universität Berlin, Berlin, Germany; 3 Centre for Biological Threats and Special Pathogens, ZBS 4: Advanced Light and Electron Microscopy, Robert Koch Institute, Berlin, Germany; 4 Department of Gastroenterology, Infectious Diseases and Rheumatology, Division of Nutritional Medicine, Charité - Universitätsmedizin Berlin, Campus Benjamin Franklin, Berlin, German; University of Texas Medical Branch, United States of America

## Abstract

Tick-borne encephalitis virus (TBEV) is one of the most important vector-borne viruses in Europe and Asia. Its transmission mainly occurs by the bite of an infected tick. However, consuming milk products from infected livestock animals caused TBEV cases. To better understand TBEV transmission via the alimentary route, we studied viral infection of human intestinal epithelial cells. Caco-2 cells were used to investigate pathological effects of TBEV infection. TBEV-infected Caco-2 monolayers showed morphological changes including cytoskeleton rearrangements and cytoplasmic vacuolization. Ultrastructural analysis revealed dilatation of the rough endoplasmic reticulum and further enlargement to TBEV containing caverns. Caco-2 monolayers maintained an intact epithelial barrier with stable transepithelial electrical resistance (TER) during early stage of infection. Concomitantly, viruses were detected in the basolateral medium, implying a transcytosis pathway. When Caco-2 cells were pre-treated with inhibitors of cellular pathways of endocytosis TBEV cell entry was efficiently blocked, suggesting that actin filaments (Cytochalasin) and microtubules (Nocodazole) are important for PI3K-dependent (LY294002) virus endocytosis. Moreover, experimental fluid uptake assay showed increased intracellular accumulation of FITC-dextran containing vesicles. Immunofluorescence microscopy revealed co-localization of TBEV with early endosome antigen-1 (EEA1) as well as with sorting nexin-5 (SNX5), pointing to macropinocytosis as trafficking mechanism. In the late phase of infection, further evidence was found for translocation of virus via the paracellular pathway. Five days after infection TER was slightly decreased. Epithelial barrier integrity was impaired due to increased epithelial apoptosis, leading to passive viral translocation. These findings illuminate pathomechanisms in TBEV infection of human intestinal epithelial cells and viral transmission via the alimentary route.

## Introduction

Tick-borne encephalitis virus (TBEV) belongs to the genus flavivirus, family Flaviviridae, mainly distributed in Europe and Asia. An infection with TBEV mostly causes flu-like symptoms such as fever, headache, nausea, vomiting and fatigue but can also result in a variety of neurological diseases including meningoencephalitis. Severity of the clinical outcome is strain-dependent and case fatality rates are ranging from less than 2% for the European strains to up to 20%–40% for some strains from Russia and the Far East [1], [2]. Worldwide, more than 10,000 cases are reported annually [3]–[5].

TBEV is mainly transmitted by the bite of an infected tick [6]. However, alimentary transmission of the virus by consumption of raw milk products from infected animals (mainly goats, sheep and cows) is also described [7], [8]. In 1951/52, the first reported milk-borne TBE outbreak took place in the Roznava district of Slovakia with at least 660 TBE cases. Since then milk-borne epidemics or single cases where reported not only from Eastern Europe but also from Austria and Germany. The number of TBE cases caused by consuming non-pasteurized milk or dairy products decreased until the early 1980s but in recent years the number of reports has increased again [5].

In Hungary, twenty-nine cases with typical TBE symptoms after consuming raw milk products and four identified TBE cases of alimentary infections were reported between 2007 and 2011 [7], [9]. Similar cases were observed in Austria, where six humans were infected with TBEV by eating infected goat cheese [10]. These outbreaks indicate that more attention has to be put on TBEV infections via the alimentary route.

While the infection route via tick bite has been elucidated in great detail, little is known about the alimentary route of infection. First experiments concerning the alimentary route were performed in the late 1950s and early 1960s in Russia and Austria. It turned out that experimental infected goats excrete TBEV up to 8 days post infection and when orally infected develop a TBEV infection with the virus detectable in the small intestine [9], [11], [12]. Furthermore, it has been demonstrated that TBEV even though it is an enveloped RNA virus, retains its infectivity in gastric juice and can pass the stomach towards the intestine [13]. Therefore, Balogh et al. [14] postulated that TBEV probably enters the organism via small intestinal M cells of the Peyer's patches which then transport the viral particles to the intestinal lymphoid tissue, but experimental evidence is missing. In another study on the tick-borne encephalitis virus group, Kenyon et al. [15] demonstrated Kyasanur Forest disease virus antigen in epithelia cells of the gut mucosa in bonnet macaques.

Other viruses, which enter their host by the alimentary route, replicate in epithelial enterocytes (coronaviruses, rotaviruses and norovirus) or can cross the mucosal barrier (poliovirus) [16], [17]. Recently, cellular entry by macropinocytosis has been described for various viruses such as influenza A, respiratory syncytial virus, or vaccinia virus [18]–[20]. Moreover, echovirus 1 is internalizing into Caco-2 cell by this mechanism, which displays many features characteristic for macropinocytosis [21]. Thus, we hypothesized that TBEV might also use macropinocytosis to enter intestinal epithelia cells. In our study, we used Caco-2 cells as a model of human intestinal epithelium, in order to analyze, whether or not TBEV can replicate in the human intestinal cells and also to unveil the cellular uptake mechanism.

## Materials and Methods

### Cell culture and viruses

Caco-2 cells (ATCC HTB-37) were grown at 37°C with 5% CO_2_ in ambient air and maintained in minimal essential medium (MEM) with 10% fetal bovine serum, 1% L-glutamine, and 1% mixture of penicillin and streptomycin. Vero E6 cells (ATCC CRL-1586) and A549 (ATTC CCL-185) cells were cultured at 37°C with 5% CO_2_ in ambient air in Dulbecco's modified Eagle's medium (DMEM) supplemented with 10% fetal bovine serum, 1% L-glutamine, 1% penicillin and 1% streptomycin. Three different TBEV strains K23, Aina and Sofjin were propagated in Vero E6 cells as published previously [22]. Viral titration was performed in A549 cells using plaque assay as described below. TBEV strain K23 was selected as a prototype for all three TBEV strains and used with a multiplicity of infection (MOI) of 1 for infection experiments unless otherwise indicated.

### Viral infection and inhibitor assays

For infection studies, Caco-2 cells were cultured in a 24 well plate and inoculated with one of the three TBEV strains (MOI of 0.1). After 1 h incubation at 37°C, unbound virus was washed off by PBS and the plate was returned to the incubator at 37°C. Cellular viral RNA was collected and viral titers in the supernatants were determined at different time points by means of quantitative real-time RT-PCR (RT-qPCR) and plaque assay as described below.

For viral inhibition assays, the pharmacological inhibitors cytochalasin D (Cyt D), nocodazole (Noc) and LY294002 (LY) were diluted in DMSO and working concentrations were as follows: Cyt D at 2 µM, Noc at 10 µg/ml and LY at 10 µM. For blockage of the cytoskeleton Cyt D was used as actin depolymerization agent [23] and Noc was used as a specific inhibitor of microtubules [24]. LY was used as inhibitor of Phosphatidylinositol-4,5-bisphosphate 3-kinase (PI3K) activation [25]. DMSO treatment (0.1% DMSO in medium) without any inhibitor was used as control. 5-(N-Ethyl-N-isopropyl)-amiloride (EIPA) was diluted in DMSO and concentrations used for the experiments were 0 µM, 25 µM and 50 µM, respectively. All products were purchased from Sigma-Aldrich (St. Louis, MO, USA). To analyze the effects of the inhibitors on TBEV entry, Caco-2 cells were pre-treated with the different inhibitors for 30 min. The cells were infected with TBEV strain K23 either in the presence or absence of the appropriate inhibitor at 37°C. After 1 h incubation the cells were washed 3 times with PBS to remove unbound viruses. To analyze virus entry, the cells were harvested for extracting total RNA followed by translation into cDNA. Thus, viral RNA was detected by RT-qPCR as described below.

### Light microscopy

Caco-2 cells were grown on coverslips in 24 well plates. Cells were infected with K23 virus and fixed at 24 h, 48 h or 72 h post infection. All samples were photographed under the light microscope (Keyence Corp, Japan).

### Immunofluorescence staining (IF)

Cells were fixed with 3.7% formaldehyde for 1 h and permeabilized with Triton-X 100 buffer (0.1% Triton-X 100 in PBS) for 10 min. After washing, samples were blocked with blocking buffer (1% bovine serum albumin (BSA) in PBS).

For detecting the envelope protein of the TBEV (E protein), samples were incubated with anti-TBEV E antibody MAB 1367 (1∶1,000) [26] and then stained with FITC-labeled anti-mouse (1∶500, Caltag Laboratories, Hamburg, Germany) or Alexa 594-labeled anti-mouse antibody (1∶200) (Invitrogen) as the secondary antibody. Cell nuclei were stained with 4′,6-diamidino-2-phenylindole (DAPI). Cells were observed and imaged using a fluorescence microscope (Keyence Corp, Japan).

For analyzing the actin filament re-arrangement induced by TBEV infection, Caco-2 cells were stained with fluorescent phalloidin (Acti-stain 488 phalloidin, Cytoskeleton Inc., Denver, USA) at 24 h post infection. For detecting co-localization of TBEV E protein with the endosomal marker proteins EEA1 or SNX5 in infected Caco-2 cells, cells were stained with the anti-TBEV E protein antibody MAB 1367 (1∶500) and with Alexa 594-labeled anti-mouse antibody (1∶200) (Invitrogen) as the secondary antibody. EEA1 or SNX5 were stained with anti EEA1 (BD Bioscience, CA, USA) or SNX5 antibody (Santa Cruz Biotechnology, CA, USA) and FITC-labeled anti-rabbit antibody as the secondary antibody (1∶500, Caltag Laboratories). Nuclei were stained with DAPI and samples were visualized by confocal laser-scanning microscopy (Zeiss LSM510, Jena, Germany).

### Ultrathin section transmission electron microscopy

For ultrathin section transmission electron microscopy, TBEV-infected Caco-2 cells were processed as descried previously [27]. Sections of epon-embedded samples were post-stained with uranyl acetate followed by lead citrate. Samples were observed using a Jeol transmission electron microscope (JEM-2100) operated at 200 kV. Photographs were taken with a CCD camera at a resolution of 2k×2k pixel (Veleta, Olympus Soft Imaging Solutions).

### Plaque assay

A549 cells were seeded in a 24 well plate and were maintained at 37°C with 5% CO_2_ overnight. 10-fold serial dilutions of the three viral suspensions were added to different wells. After 1 h incubation each well was filled with 500 µl carboxymethylcellulose (CMC) overlay medium (1.6% CMC in DMEM). The plates were continually maintained in the CO_2_ incubator at 37°C. After 4 days, each well was fixed in 3.7% formaldehyde for 1 h, and then the cells were stained with Naphthalene Black (1 g of naphthol blue black, 13.6 g of sodium acetate, 60 ml of glacial acetic acid and up to 1000 ml with bi-destillated H_2_O). Plaques were counted and expressed as plaque-forming units/ml (PFU/ml). The viral titers were calculated by the equation:
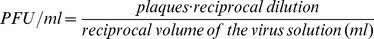



### RNA isolation and RT-qPCR

Total RNA from Caco-2 cells was prepared according to Manufacturer's instructions using the Qiagen RNeasy mini kit (Qiagen, Hilden, Germany). For cDNA synthesis, the same amount of RNA was added in a final reaction volume of 20 µl using the Superscript II kit (Invitrogen, Karlsruhe, Germany). The mixture was pre-treated for 10 min at 65°C and cooled down on ice for 5 min. Then the reverse transcription reaction was performed on a thermoblock cycler for 60 min at 37°C and 10 min at 93°C. 2 µl cDNA was detected by quantitative RT-PCR as described previously [22]. For TBEV quantification the viral NS1 gene was detected.

### Transport assay of TBEV in Caco-2 monolayers

Caco-2 cells were seeded in permeable PCF-filter cell culture inserts with an area of 0.33 cm^2^ and with a pore size of 0.4 µm (Millipore, Darmstadt, Germany) and were grown for three weeks. Media was replaced every 2 days. Experiments were performed with cells showing a transepithelial electrical resistance (TER) above 300 Ω·cm^2^. After treatment of Caco-2 monolayers with the TBEV (MOI of 1) for 1 h, fresh culture medium was replaced and TER of each transwell was determined every 24 h using an epithelial volt ohmmeter with a pair of chopstick electrodes (EVOM, World Precision Instruments, FL, USA). Untreated monolayers were used as negative controls. In the course of virus infection, the same aliquots of medium were collected from the lower chambers at different time points as indicated. TBEV in the medium were detected using the RT-qPCR method as described above.

### FITC-Dextran fluid uptake assay

Caco-2 cells were seeded on glass coverslips in a 24 well plate until getting confluent and infected with TBEV for 4 h. Then Caco-2 cells were incubated with FITC-dextran (Molecular Weight 70 000 Da, Sigma-Aldrich) at a final concentration of 0.5 mg/ml in the absence or presence of TBEV. After 30 min cells were washed 3 times with PBS and subsequently fixed. Simultaneous acquisition of FITC fluorescence emission and transmitted light from all samples was done by confocal laser-scanning microscopy. Vesicle count was done by ImageJ particle analysis tool with fluorescence intensity threshold of Caco-2 monolayers where no Fdx was added.

### Cell viability

MTT assay was used to analyze cell viability. Caco-2 cells were cultured into a 96 well culture plate for 2 days. After removal of the cell culture medium, cells were infected with TBEV (MOI of 1). Virus free cells were used as control. At 48 h or 120 h post infection, medium was replaced with 20 µl of 3-(4,5-Dimethylthiazol-2-yl)-2,5-diphenyl tetrazolium bromide (MTT) (5 mg/ml) solution and cells were further incubated at 37°C for 4 h. Then the MTT solution was removed and 200 µl of DMSO was added and gently swirled. After formazan crystals were dissolved, the colorimetric reaction was measured at 570 nm using a spectrophotometer (Tecan Group Ltd., Maennedorf, Switzerland).

### Epithelial apoptosis

TUNEL assay (deoxynucleotidyl transferase-mediated deoxyuridine triphosphate nick-end labeling, Roche Diagnostics, Mannheim, Germany) was used to analyze apoptosis induction by TBEV infection. Coverslips with infected Caco-2 cells were fixed with 3.7% formaldehyde at 48 h or 120 h post infection. After washing with PBS, the samples were permeabilized using TritonX buffer (0.1% Triton-X100 in PBS) for 10 min and washed with PBS 3 times. The TUNEL assay was performed following the manufacturer's recommendations. Cell nuclei were counterstained with DAPI. All samples were examined under fluorescence microscope (Keyence Corp.) in low-power fields.

### Statistical analysis

All results are shown as mean ± standard error of the mean (SEM). Statistical analyses were performed using Prism5 software (GraphPad, San Diego, Canada). Differences between treatment and control groups were evaluated using the Student's t-test. A *P*-value of <0.05 was considered statistically significant.

## Results

### TBEV replication in human intestinal Caco-2 cells

**Figure 1:**
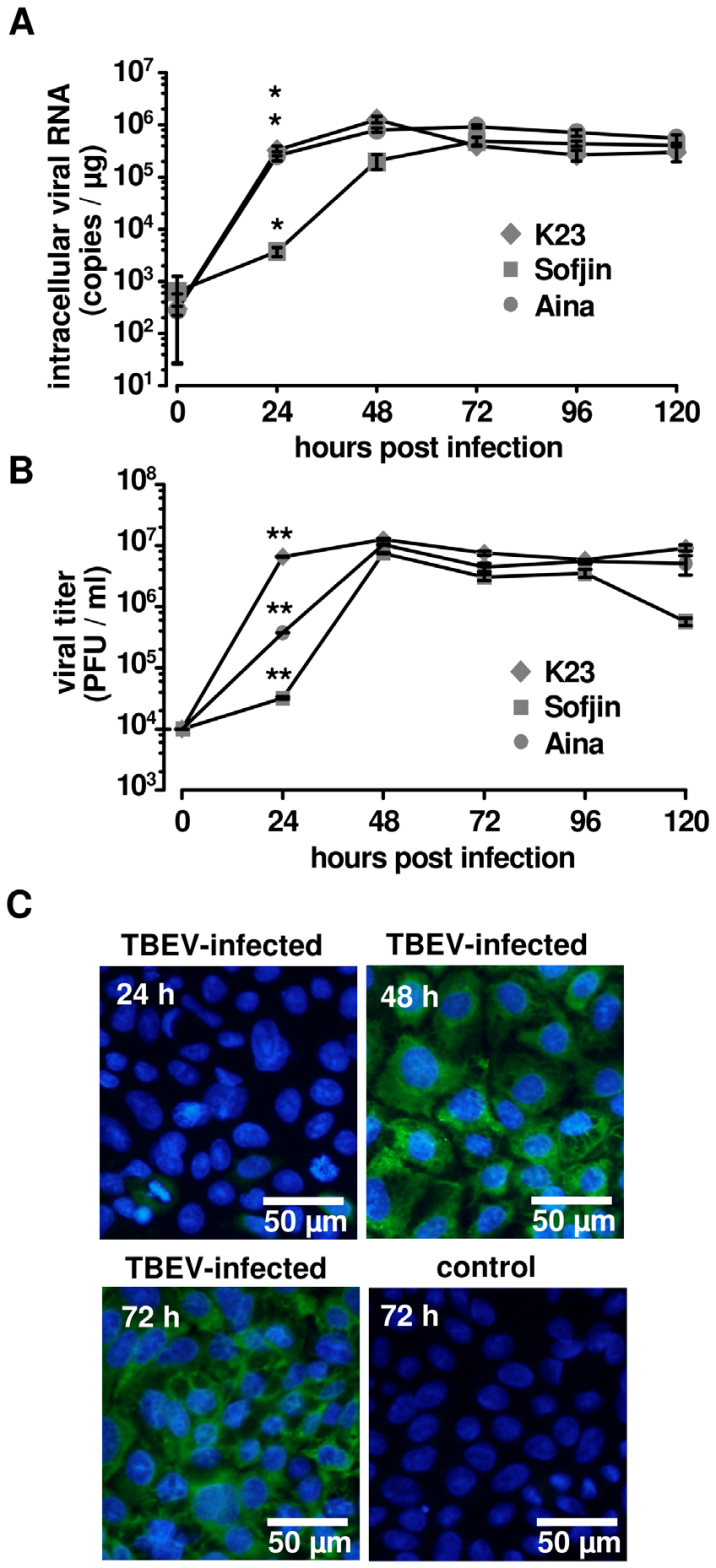
Caco-2 cells are susceptible to TBEV infection. Human cells infected with TBEV strains K23, Sofjin, and Ania at an MOI of 0.1. Viral supernatant and intracellular viral RNA were harvested at 24(**A**) Intracellular TBEV RNA copy numbers, measured by RT-qPCR. (**B**) Viral titers in the supernatant determined by plaque assay, n = 3; *P<0.05, **P<0.01 to initial virus titer in Student's *t* test. (**C**) Immunofluorescence assay of TBEV-infected Caco-2 cell monolayers. Caco-2 cells infected with TBEV K23 strain were fixed at different time points and subjected to immunofluorescence assay. TBEV E (green), nuclei (blue, DAPI = 4′-6-diamidino-2-phenylindole dihydrochloride). One representative image of a triplicate is shown.

Caco-2 cells were challenged with TBEV strain K23, Sojin, or Aina at a multiplicity of infection (MOI) of 0.1. Intracellular viral RNA was analyzed by RT-qPCR. Viral copy numbers of the three strains increased at the first day of infection, peaked at day 2 post infection (p.i.) and persisted in high amounts in the cells up to day 5 p.i. (Figure 1A). The amount of released TBEV particles in cell culture supernatant was highest on day 2 p.i. for all 3 TBEV strains (Figure 1B). The virus titer in the apical cell supernatant increased by 3 log numbers between day 1 and 2. We further monitored TBEV infection in Caco-2 cells with TBEV strain K23 by immunofluorescence microscopy. As shown in Figure 1C nearly 100% of the cells were found TBEV-positive at 48 h p.i., while only few cells were positive at 24 h p.i. This rapid virus spread between cells confirmed that TBEV replication is efficient in human intestinal Caco-2 monolayers and that the cells in general are susceptible to TBEV infection. Beside the apical infection of confluent Caco-2 monolayers we detected an efficient replication also of subconfluent Caco-2 cells (data not shown).

### Vacuolization by TBEV infection in Caco-2 cells

**Figure 2:**
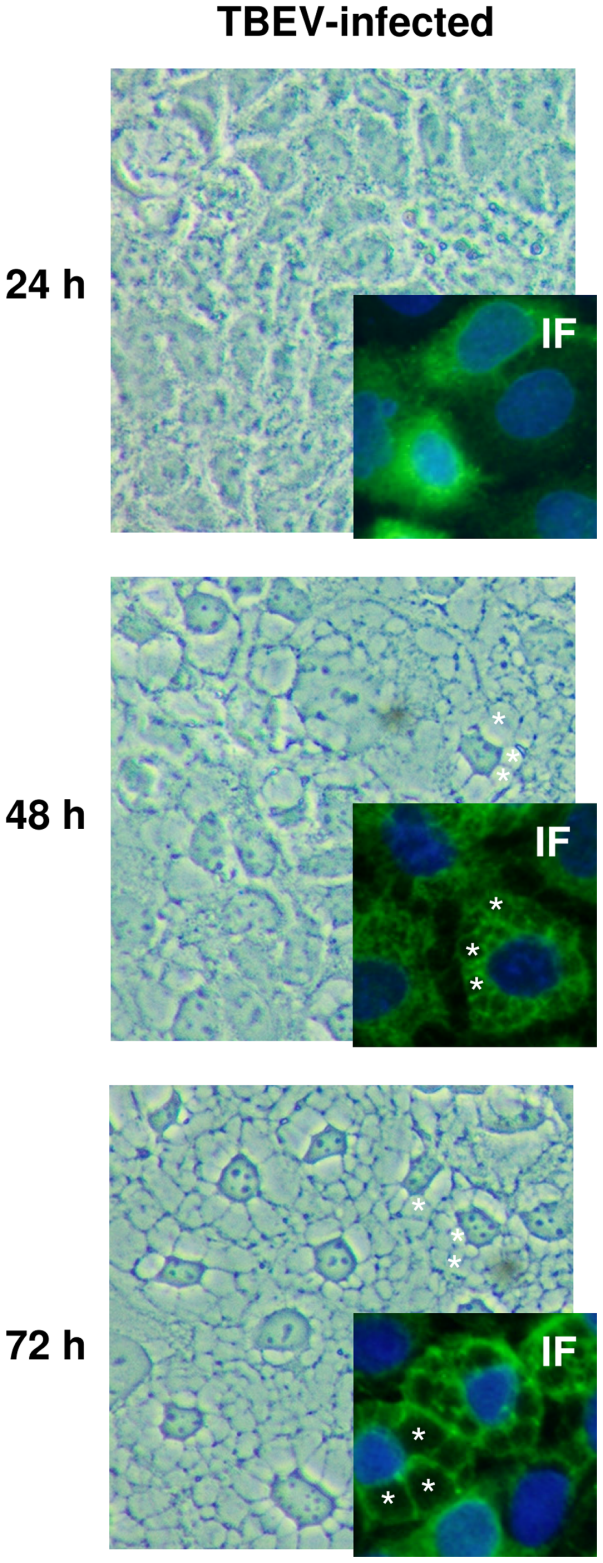
Vacuolization induced by TBEV infection. Caco-2 cells were infected with TBEV K23 virus. Cellular morphological changes and vacuolization were monitored by light microscopy. Caco-2 cells were infected with TBEV strain K23 and fixed at 24 h, 48 h and 72 h. Cells were observed with the 40× objective (400× total magnification). Details of cytoplasmic vacuolization are visualized by immunofluorescence (IF) microscopy. 3 representative vacuoles are indicated by white asterisks in each sub-image. Samples were incubated with anti-TBEV E antibody and then stained with secondary anti-mouse antibody conjugated to FITC (green). Nuclei were stained with DAPI (blue).

In the course of TBEV infection in Caco-2 cells strong vacuolization was found, whereas morphological changes such as aggregation and shrinkage of cells or detachment of the monolayer were not observed at 48 h p.i. (Figure 2). TBEV-induced vacuolization in infected Caco-2 cells was detected by immunofluorescence microscopy using anti-TBEV E monoclonal antibody at 24 h, 48 h, and 72 h p.i. (Figure 2).

### Ultrastructural analysis of TBEV-infected Caco-2 cells

**Figure 3:**
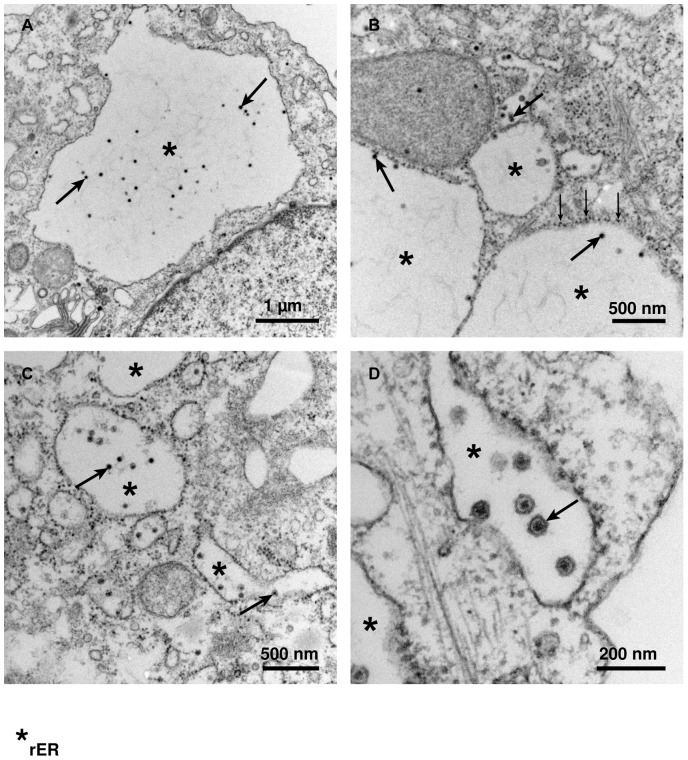
Ultrastructural analysis of TBEV-infected Caco-2 cells by ultrathin section transmission electron microscopy. All photographs were taken at 12(rER) containing TBEV are indicated by asterisks and ribosomes of the rER are indicated by small vertical arrows. (A) bar  = 1 µm, (B) bar  = 500 nm, (C) bar  = 500 nm, (D) bar  = 200 nm.

We analyzed the ultrastructural changes induced by TBEV infection in Caco-2 cells using ultrathin section transmission electron microscopy. A dilatation of the rough endoplasmic reticulum (rER) and presence of virus particles in rER cisternae were the first ultrastructural signatures of virus replication. At later stages large membrane-bound caverns in the cytoplasm contain most of the observed virions. The cavern membrane was coated with ribosomes indicating that it derived from the rER (Figure 3).

### Cytoskeletal changes and inhibition of virus entry

**Figure 4:**
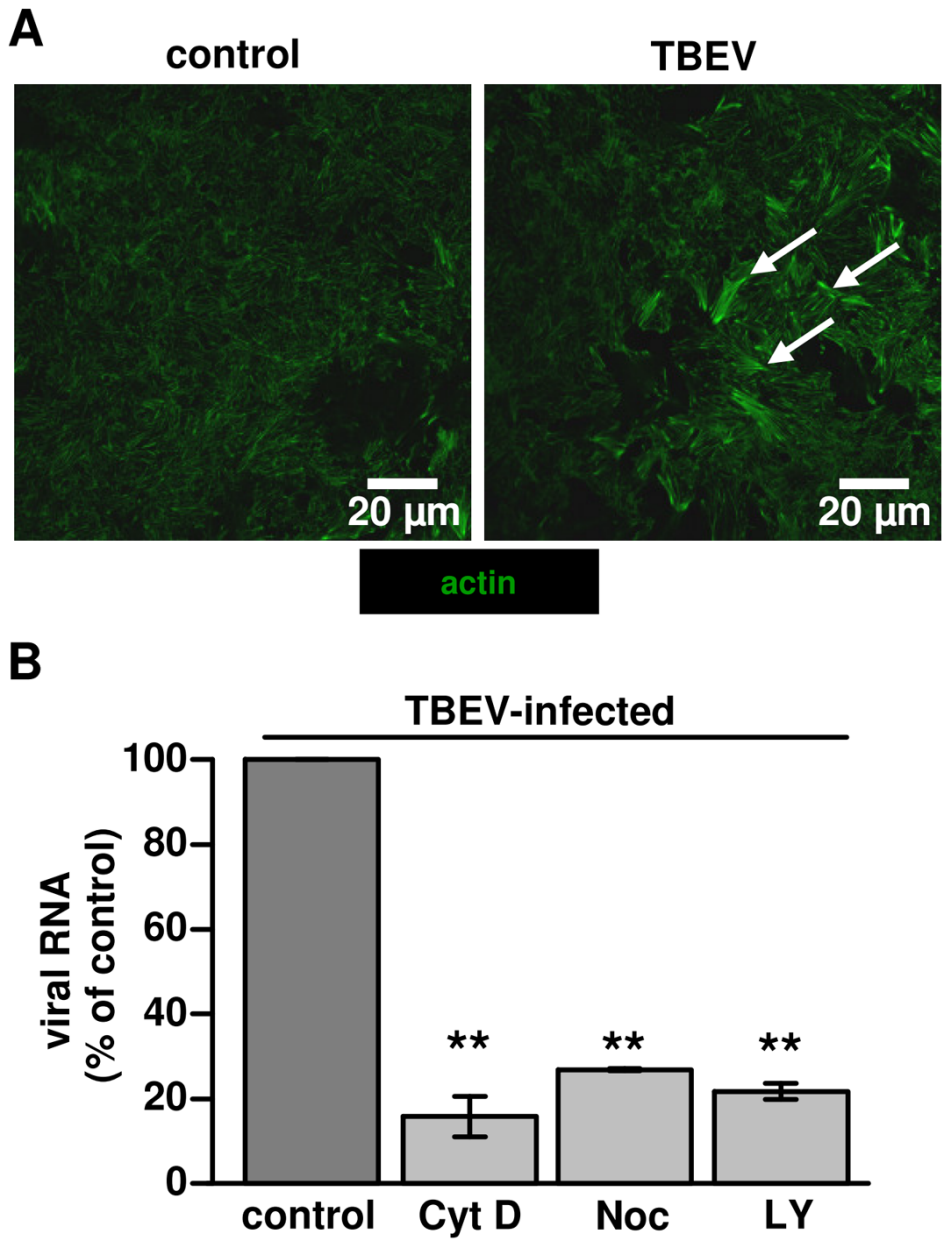
Cytoskeletal integrity is important for TBEV infection in Caco-2 cells. (**A**) Actin re-arrangements following TBEV infection. Cells infected with TBEV strain K23 were fixed at 24 h. Samples were stained for actin microfilament and the apical cell-domain (perijunctional cytoskeleton) was visualized by fluorescence microscopy with Acti-stain 488 phalloidin. Non-infected cells were used as controls. White arrows indicate representative areas of more condensed actin filaments in the right image. (**B**) Inhibition of TBEV cell entry by blocking microfilaments. Caco-2 cells were treated with cytochalasin D (Cyt D), nocodazole (Noc) or LY294002 (LY) for 30 min. DMSO treated Caco-2 cells were used as control. After treatment with inhibitors, cells were incubated with TBEV strain K23 for 1 h. Virus entry was monitored by RT-qPCR, n = 3; **P<0.01 to control in Student's t test.

Initial cytoskeletal changes were observed 24 h p.i. The actin cytoskeleton showed a general re-arrangement and more condensed microfilaments were observed than in non-infected controls (Figure 4A). To test the response of the cytoskeleton to virus entry, we conducted inhibition experiments with inhibitors of cytoskeletal actin (cytochalasin D), microtubules (nocodazole) or autophagy/endocytosis via PI3-Kinase (LY294002). All inhibitors induced a reduction in intracellular virus entry (Figure 4B). Since actin is required for the formation of plasma membrane ruffles in macropinosome formation as well as for trafficking of macropinosomes into the cell [28], we hypothesized that TBEV entry is mediated by a macropinocytosis-like mechanism.

### TBEV entry into Caco-2 cells shows characteristics of macropinocytosis

**Figure 5:**
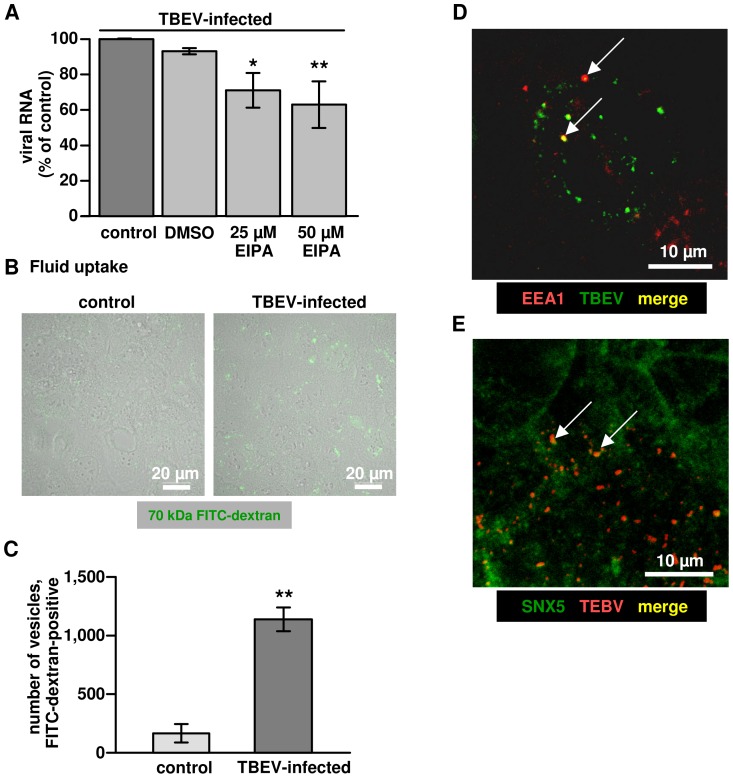
Characteristics of macropinocytosis in TBEV infected Caco-2 cells. (**A**) Impaired TBEV entry by EIPA. Dose-dependence of EIPA-induced inhibition of TBEV entry. Caco-2 cells were pre-treated with EIPA for 30 min, followed by incubation with TBEV in the presence of the inhibitor. After 1 h, virus entry was monitored by RT-qPCR. n = 4; *P<0.05, **P<0.01 to DMSO control in Student's t test. (**B**) Fluid uptake. Accumulation of intracellular FITC-dextran (green) induced by TBEV infection. Caco-2 cells were treated with TBEV strain K23 for 1 h and then washed with PBS. Subsequently, cells were incubated with FITC-labeled dextran (1 mg/ml). After 4 h, cells were washed, fixed and observed by confocal microscopy. One representative image of a triplicate is depicted. (**C**) Accumulation of dextran in cells was analyzed by counting the total number of intracellular FITC-dextran-containing vesicles in a low power field using ImageJ. **P<0.01 (n = 3). (**D**) Immunofluorescence microscopy. TBEV co-localization (as merge in yellow, indicated by arrows) with early endosomal antigen-1 (EEA1) or (**E**) Sorting nexin-5 (SNX5) after virus entry. Cells were fixed and stained for TBEV anti-E protein and EEA1 or SNX5 with primary antibodies, followed by secondary antibodies as indicated in the image. A representative image (63× objective) is shown. Yellow dots as merge indicate TBEV particles in co-localization with EEA1 or SNX5.

The use of amiloride (data not shown) and its more potent derivative EIPA (5-(N-Ethyl-N-isopropyl)-amiloride) block the epithelial sodium channel (ENaC) as well as dose-dependently several other Na^+^/H^+^ antiporters. EIPA has often been used as a hallmark inhibitor that specifically inhibits endocytosis via the macropinocytic pathway [29]. As shown in Figure 5A, TBEV entry into Caco-2 cells is inhibited by EIPA treatment in a dose-dependent manner. One characteristic of macropinocytosis is the nonselective uptake of large amounts of extracellular solutes [28]. To further investigate the involvement of macropinocytosis in TBEV entry, the uptake of soluble FITC-labeled dextran (Fdx) into Caco-2 cells was monitored. Fdx has often been applied as a morphological marker for macropinosomes and is used in fluid uptake assays. We found that TBEV infection slightly increased the uptake of Fdx into Caco-2 cells from 166±79 vesicles and a total particle area of 5±2 µm^2^ in mock control versus 1138±101 vesicles with a total particle area of 60±4 µm^2^ (p<0.01 and P<0.001 respectively; n = 3) in a high-power field of 135 µm^2^ in TBEV infected cells (Figure 5B). The average particle size of 0.04±0.01 µm^2^ in control was not different from Fdx vesicles in TBEV-infected Caco-2 monolayers with 0.06±0.01 µm^2^ particle size (n = 3, *n.s.*). In addition, Early Endosome Antigen-1 (EEA1) was shown to be a marker of newly formed macropinosomes and mediated virus entry in cultured cells [30], [31]. The protein sorting nexin-5 (SNX5) mediates macropinosome formation and is involved in its maturation [32]. For this reason we analyzed co-localization of TBEV with endogenous EEA1 or SNX5 in Caco-2 cells. Figure 5D and E show a co-localization of TBEV E protein 24h p.i. with EEA1 or SNX5, respectively (See also Z-stack Figure S3 and S4 as well as Video S1 and S2). Taken together, these findings indicate macropinocytosis as a mode of TBEV entry and internalization.

### Translocation of TBEV via the paracellular pathway in the late phase of infection

**Figure 6:**
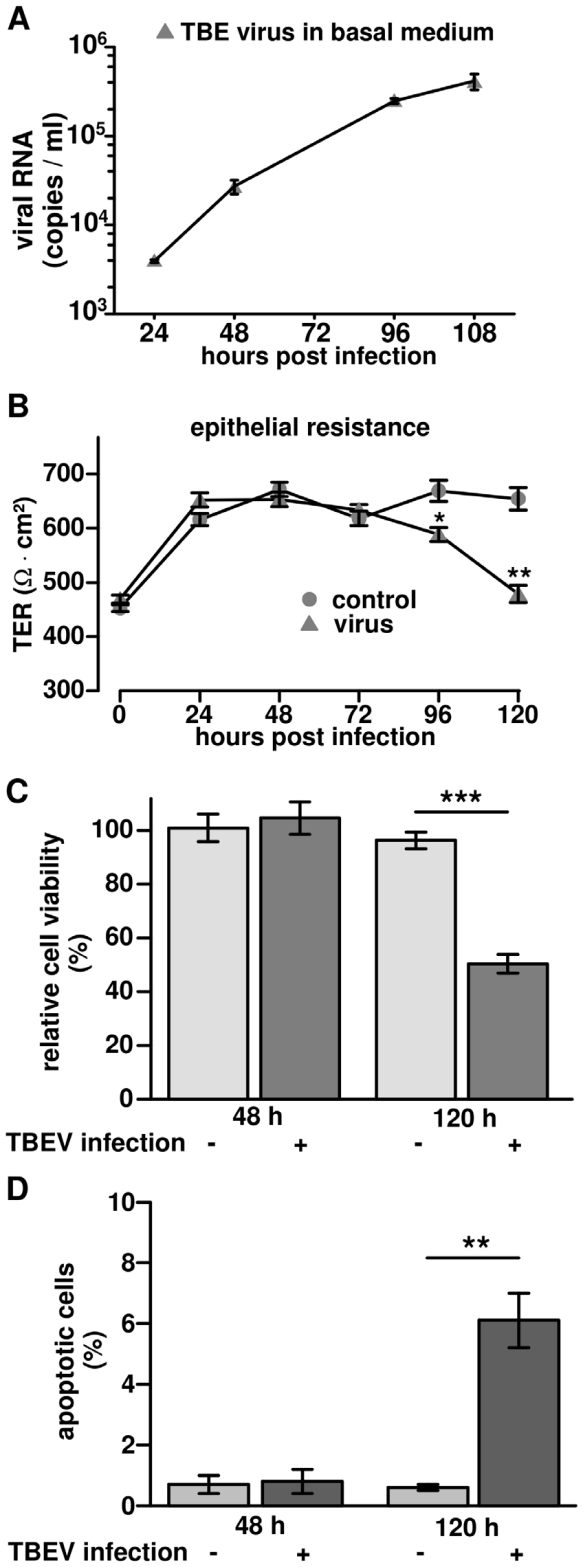
Translocation of TBEV through Caco-2 monolayers without affecting transepithelial electrical resistance (TER). (**A**) Virus amount in basal medium. Polarized Caco-2 monolayers grown on permeable supports were infected with TBEV strain K23 from the apical surface. Basal medium was harvest at different time points and viral RNA in each sample was detected by RT-qPCR. The data were displayed as mean with standard deviation. (**B**) Transepithelial electric resistance (TER) measurements during TBEV infection. Polarized Caco-2 monolayers, grown on permeable supports, infected with TBEV K23 (circles) from the apical surface. Non-infected cells served as controls (triangles). TER values were measured from 0 h to 120 h post infection. n = 5, *P<0.05, **P<0.01 to control in Student's t test. (**C**) Cell viability during TBEV infection. Caco-2 cells were infected with TBEV strain K23 at an MOI of 1 and cell viability was analyzed by MTT assay. Cell viability was measured and calculated as a percentage of non-infected control cells. Data were expressed as mean ± standard error of the mean. (**D**) Analysis of TUNEL-positive cells. Confluent Caco-2 cells were infected with TBEV strain K23 and apoptosis was detected by a terminal deoxynucleotidyl transferase-mediated deoxyuridine triphosphate nick-end labeling (TUNEL) at 48 h and 120 h post infection. The ratios of TUNEL-positive cells to all cells were analyzed in 4 low-power fields from 3 independent samples of each group. **P<0.01.

During the transmission of TBEV by the oral route, virus may be released into the circulation after crossing the intestinal epithelium. To test this hypothesis, viruses were added to polarized Caco-2 cell monolayers that were grown on permeable filter supports for 3 weeks. Virus incubation was performed for 1 h. Virus release into the basal medium was determined by measuring viral RNA copies over 5 days. As shown in Figure 6A, the amount of TBEV RNA copies in basal medium persistently increased in the course of infection, although TBEV was not detectable in the basal medium at 0 h post infection. Simultaneously, TER was recorded, in order to determine, whether or not TBEV affects epithelial barrier function. Figure 6B shows that TER remained stable for 72 h and decreased 4 days after infection.

To exclude that lesions due to TBEV-induced cell death caused the translocation of the virus into the basal medium, cell viability was monitored by MTT assay. No change in cell viability was observed in the early phase of infection (until 2 days p.i.). However, 5 days p.i. cell viability decreased (Figure 6C).

To corroborate these findings, TUNEL assays were performed to assess the apoptosis ratio in TBEV infected Caco-2 cells (Figure S1). 2 days p.i. the percentage of apoptotic cells was close to 0 and not different from untreated controls, but 5 days p.i. around 5% apoptotic cells were found (Figure 6D). These results suggest that TBEV significantly accelerated apoptosis in Caco-2 cells in the late phase of infection. Thus, in the early phase of infection (up to 2 days p.i.) no evidence for apoptosis induction was obtained and TER of the Caco-2 monolayers remained unaffected indicating an intact epithelial barrier. Therefore, virus translocation in the early phase of infection points to virus transcytosis. Whereas the decline in the integrity of the monolayer after 4 days p.i., as measured by a reduction in TER (Figure 6B) and an increase in apoptosis ratio (Figure 6D) as well as cytoskeletal (Figure 4A) and subsequent tight junction changes (Figure S2, Video S3), suggests an additional route of virus translocation via the paracellular pathway.

## Discussion

Tick borne encephalitis virus is mainly transmitted by infected ticks. After a bite of an infected tick, the virus invades the human central nervous system. A variety of studies on the neural pathogenesis of TBEV in vivo and in vitro were shown [33]–[35]. Recently, some TBE cases were caused by consuming raw milk from infected animals [7], [9]. However, the details of TBEV transmission by the alimentary route are not well known. Therefore, we used Caco-2 cells as an in vitro model to display the interaction between TBEV and human intestinal epithelial cells.

We found that TBEV infected and replicated efficiently in human intestinal epithelial cells. The Caco-2 cell model, if grown for 3 weeks, is a suitable infection model for the small intestine, because it develops small intestine-like properties e.g. low transepithelial electrical resistances or expression of SGLT-1 sugar transporters [36], [37]. In our experiments TBEV replicates rapidly and after 2 days p.i. nearly all cells were infected although infectious dose was relatively low (MOI 0.1). In the early phase of infection up to 48 h the cell monolayers' integrity remained stable as indicated by an unchanged TER, no induction of epithelial apoptosis and no obvious tight junction changes in IF stainings (data not shown). This is in contrast to other viruses causing gastrointestinal (GI) symptoms e.g. experimental rotavirus infection in Caco-2 monolayers caused a rapid decrease of TER and a massive tight junction dysregulation within the first 24 h [38].

The second important finding of this study was that macropinocytosis is an endocytic pathway in TBEV infection. Virus trafficking via macropinosomes was recently described for a growing number of viruses from other families such as echovirus [39], lentiviral HIV [40], or as reviewed for Vaccinia virus, Adenovirus 3, Coxsackievirus B, and Herpes simplex virus 1 etc. [28]. Several lines of evidence indicate that the TBEV internalization by Caco-2 cells is associated with macropinocytosis: (i) TBEV was detected in mid-sized vesicles of approximately 200 to 500 nm by EM in Caco-2 cells. These observations also revealed the virus particles probably assemble in the rER [41]. (ii) Intracellular trafficking of TBEV containing vesicles was mediated by e.g. SNX5 signaling, which regulates the formation and maturation of macropinosomes [23]. Also EEA1 presented evidence for early endosomes in co-localization with TBEV. (iii) Inhibition of actin- or microtubule-dependent cytoskeleton polarization blocked virus particle trafficking and the inhibition of PI3K signaling also blocked virus uptake. (iv) Inhibition experiments with EIPA and fluid uptake assays of infected Caco-2 cells provide further evidence for a macropinocytosis mechanism. All these findings support the hypothesis that uptake of viral particles is mediated by the process of macropinocysis. Since the reduction in virus endocytosis in these experiment was partial, the evaluation of e.g. clathrin-mediated endocytosis by other specific inhibitors is up to further investigations.

In our study we showed how the flavivirus TBEV infect intestinal Caco-2 cells. We now postulate that TBEV transmission and translocation into the organism can occur via the small intestine, but this remains to be experimentally confirmed by an animal infection model. Although some cases of TBEV infection of lab workers by aerosols were described, when cell culture flask containing high amounts of virus were broken in the incubator [42], TBEV transmission via the respiratory tract or oropharyngeal epithelial cells needs to be further investigated. In addition, oral experimental infection of animals resulted in a TBEV infection after an incubation time of several days [11], [12]. TBEV can resist to gastric juice that showed the possibility of alimentary TBEV infection [13]. Furthermore, other viruses such as HIV and EBV can induce persistent infections after oral transmission in animal models [43], [44]. Therefore, it is reasonable that virus translocation via the small intestine is a possible route when humans consume unpasteurized milk products. Milk-borne infections and translocation across tight human epithelial barriers were also reported for other virus, as e.g. Human T-cell leukemia virus (HTLV-1) [45]. Furthermore, Human immunodeficiency virus (HIV) transmission from maternal milk was shown [46]. Milk as a vehicle for pathogen transmission is often observed and milk or its products protect pathogens to survive in the gastric acidic environment in order to get access to enterocytes. As the buffer capacity of milk and milk products is quite high, antibacterial or antiviral activity of the gastric juice is lowered when consuming milk, especially in young children or person with low gastric secretion [47]. Therefore, the gastric passage of pathogens (even in case of the gastric pathogen *Helicobacter pylori*) depends on the susceptibility of the hosts as well as on the type of the diet [47].

In our experimental infection model, the beginning structural changes of the cytoskeleton and vacuolization in the early phase lead to further developed pathological changes in the late phase of infection. Although we cannot completely exclude virus translocation e.g. via apoptotic leaks, we found indications that the epithelium was not hampered in its integrity up to 48 h p.i. Thus, we conclude that virus particles were released via exocytosis. However, after prolonged incubation time up to 5 days p.i. apoptosis ratio was massively increased in infected cell monolayers. Concomitantly, TER decreases after 4 days and was lowered to the half of the initial value at day 5. Therefore, paracellular virus translocation through the epithelial barrier may occur in the late phase of infection in the leaky epithelium. The induction of apoptosis by cytokine production, as shown for cytokine-containing supernatants of HIV-infected cells may also contribute to the barrier defect [48]. Moreover, we found additional pathological changes in late TBEV infection like the subcellular distribution of tight junction proteins in single areas of infected Caco-2 monolayers that can promote virus translocation via the paracellular pathway (Figure S2). These kind of focal barrier defects were prominently observed in infection models of bacterial enteric pathogens e.g. *Yersinia enterocolitica* [49], *Campylobacter concisus* [50] or *Escherichia coli* 536 [51]. The drop in TER by TBEV can be assigned to the massive induction of apoptosis in the late phase of infection, but also re-distribution of barrier-forming tight junction proteins may contribute to the epithelial barrier defect. The lowered epithelial barrier function together with apoptosis induction were induced by the virus directly and/or by the host response to the infection (e.g. cytokine induction) likewise shown for infection of the small intestine by other viruses, e.g. astrovirus or HIV [48], [52]. Pathological apoptosis induction can influence TER and increase permeability of the epithelium for macromolecules up to 4 kDa [50], [53]. Thus, a passive uptake mechanism into the organism for virus translocation and on the other hand the loss of solutes and water (diarrhea) is supposedly a further pathogenic features of TBEV infection. Similarly, the pathogenic mechanisms in the human small intestine during norovirus infection could be described by epithelial apoptosis induction and tight junction dysregulation [54]. Moreover, the norovirus p20 protein showed interference with epithelial restitution mechanisms when stably transfected into HT-29/B6 colon cells [55]. Likewise tight junction disruption caused by the capsid of the West Nile virus was found in Caco-2 monolayers [56]. From HIV infection it is known that the enteric immune cells were the site of virus progeny and that the HIV causes GI symptoms per se in the acute phase of infection (HIV enteropathy), thereby both apoptosis induction and tight junction changes contribute to the diarrhea [57]. Thus, it is supposable that the GI tract may also serve as the site for TBEV propagation and dissemination.

As TBEV can translocate via the transcellular as well as the paracellular pathway, the entry of the virus into the organism may lead to a systemic infection and subsequently infection of the nervous system. It was demonstrated that TBEV lead to breakdown of the blood-brain barrier in experimental infected mice [35]. Moreover, another report showed that TBEV infection of rat astrocytes did not influence their cell viability [34]. These studies, together with our findings, indicated that both transcellular as well as paracellular translocation of the virus can occur during infection. The mechanisms by which the viruses perturb the intestinal epithelial barrier by transcytosis and paracellular translocation (e.g. over apoptotic leaks) is also supposable for virus translocation through the endothelial blood-brain barrier. The tight junction can be involved in virus endocytosis and replication. For example the Hepatitis C virus exploits the tight junction proteins occludin and claudin-1 as receptors for cell entry into liver cells [58]. In our cell model we did not find any co-localization of TBEV and tight junction or other apical membrane compartments such as lipid-rafts (data not shown), thus a cellular receptor for TBEV entry remains unknown. As Melik and co-workers reported that PDZ-domains might be important for TBEV replication and assembly [59], it is supposable that PDZ-motives of tight junction proteins (e.g. occludin) would be used by the virus and thereby tight junctional dysregulation may be induced. However, tight junction dysregulation by the virus has to be determined separately from apoptosis induction, which can facilitate tight junction changes alone. For further research, the cellular defense mechanisms (clearance of virus particles) in low-dose TEBV-infected epithelial cells are worthwhile to proceed. In preliminary experiments low infectious doses did not affect epithelial integrity nor resulted in high replication rates as seen with MOI above 0.1 (data not shown). Therefore, a possible transmission of TBEV between the cells via actin filament rearrangement should be considered for upcoming observations which seem to play an important role in TBEV infection [60]. Taken together, TBEV is able to translocate through the intestinal epithelial barrier providing evidence that virus infection can occur via the alimentary route. In Caco-2 cell monolayers, TBEV entry into intestinal epithelial cells is mediated by macropincytosis and replication of virus leads to high virus titers in apical and basal compartments. Future studies should confirm the findings on barrier breaking properties of TBEV infection on epithelial and endothelial borders in animal models and clinical observations.

## Supporting Information

Figure S1 **TUNEL assay in TBEV infected Caco-2 cells, supplementary to** **Figure 6D** **.** Cellular apoptosis induced by TBEV infection. TUNEL assay in TBEV infected Caco-2 cells. Cells were infected with TBEV and apoptosis was detected by TUNEL (red) at 48 h and 120 h post infection. Cells were observed with the 20× objective (200× total magnification). Nuclei were stained with DAPI (blue). Micrographs were taken by fluorescence microscopy.
(TIF)

Figure S2 **The effects of TBEV on tight junction changes may also contribute the drop in TER.** Representative tight junction protein ZO-1 distribution and F-actin as cytoskeletal marker were stained in TBEV-infected and non-infected Caco-2 cells to display structural correlates to the electrophysiological findings. (A) ZO-1 and F-actin were disrupted by TBEV infection. Cells were fixed and stained for ZO-1 with primary antibodies and secondary anti-Rabbit Alexa Fluor 594 (red), TBEV E monoclonal antibody and anti- mouse conjugated with FITC (green). F-actin (white) stained with Atto-Phalloidin 647N (Sigma-Aldrich). Nuclei stained with DAPI (blue). Micrographs were taken by confocal microscopy. (B) Corresponding image of (A) as Z-stack in XY-plane.
(TIF)

Figure S3 **Co-localization of TBEV and EEA1.** The infected cells were observed using confocal microscopy. The Z-stack image shows the virus co-localizes with EEA1 as yellow dots in XY-plane.
(TIF)

Figure S4 **Co-localization of TBEV and SNX5.** The Z-stack images in XY-plane were taken using confocal microscopy. The left image shows the virus co-localizes with SNX5 as yellow dots. In the right control image SNX5 is evenly distributed.
(TIF)

Video S1 **Co-localization of TBEV and EEA1.** Infected Caco-2 cells were observed using confocal microscopy and 3D video was created with Carl Zeiss LSM Image Examiner software. The moving 3D image shows the virus in co-localization with EEA1 as yellow dots.
(MOV)

Video S2 **Co-localization of TBEV and SNX5.** The moving 3D image shows the virus in co-localization with SNX5 as yellow dots.
(MOV)

Video S3 **Tight junction changes induced by TBEV infection.** The moving 3D image (from Figure S2) shows that TBEV rearrange ZO1 distribution in affected regions of Caco-2 monolayers.
(MOV)
