# Supplementary figures and images for: Tick-Borne Encephalitis Virus Replication, Intracellular Trafficking, and Pathogenicity in Human Intestinal Caco-2 Cell Monolayers
Source: PLoS One. 2014 May 12;9(5):e96957. doi: 10.1371/journal.pone.0096957 (PMC4018392; doi:10.1371/journal.pone.0096957)

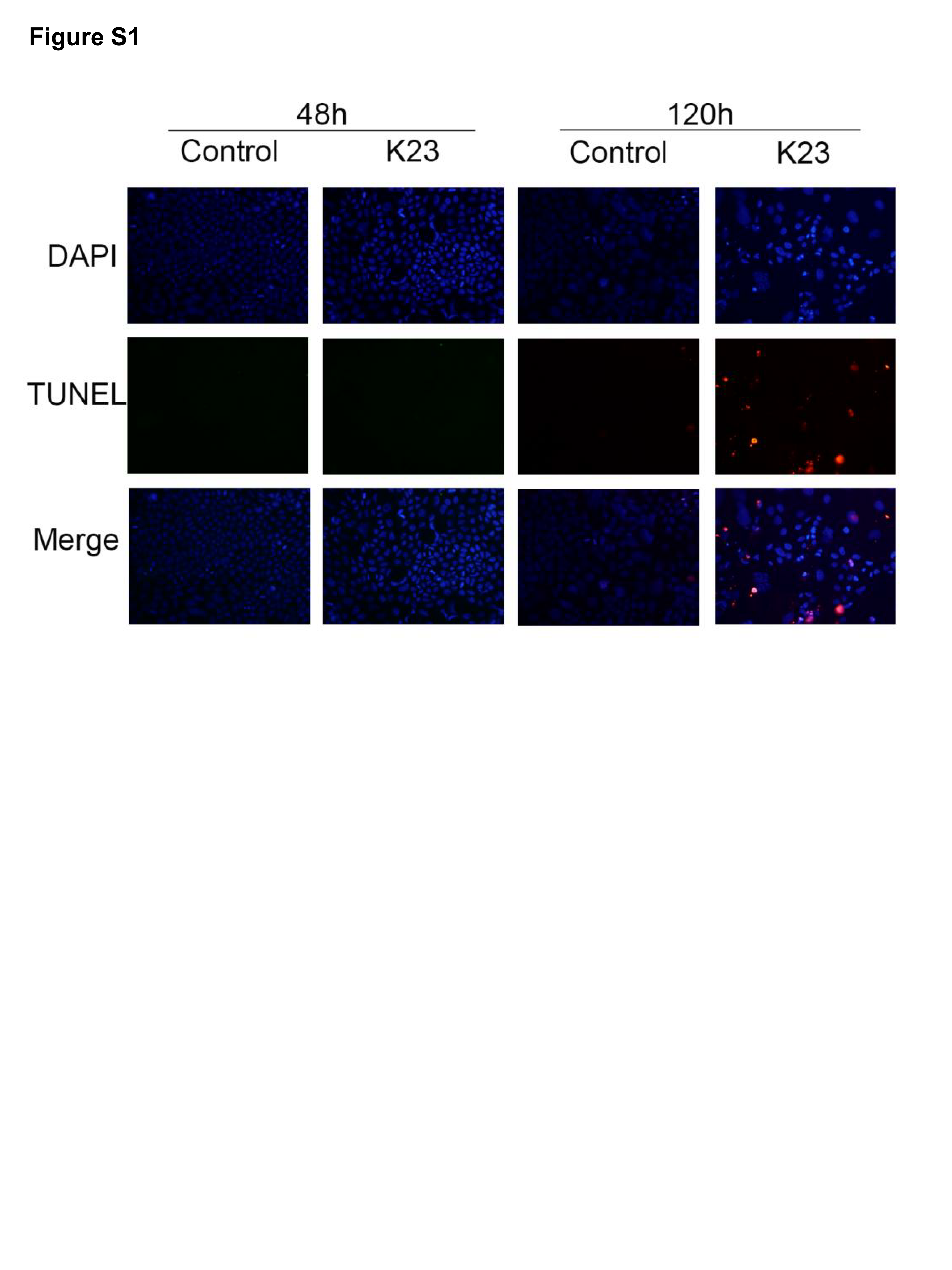

Supplement: Figure S1 — TUNEL assay in TBEV infected Caco-2 cells, supplementary to Figure 6D . Cellular apoptosis induced by TBEV infection. TUNEL assay in TBEV infected Caco-2 cells. Cells were infected with TBEV and apoptosis was detected by TUNEL (red) at 48 h and 120 h post infection. Cells were observed with the 20× objective (200× total magnification). Nuclei were stained with DAPI (blue). Micrographs were taken by fluorescence microscopy. (TIF) [file pone.0096957.s001.tif]

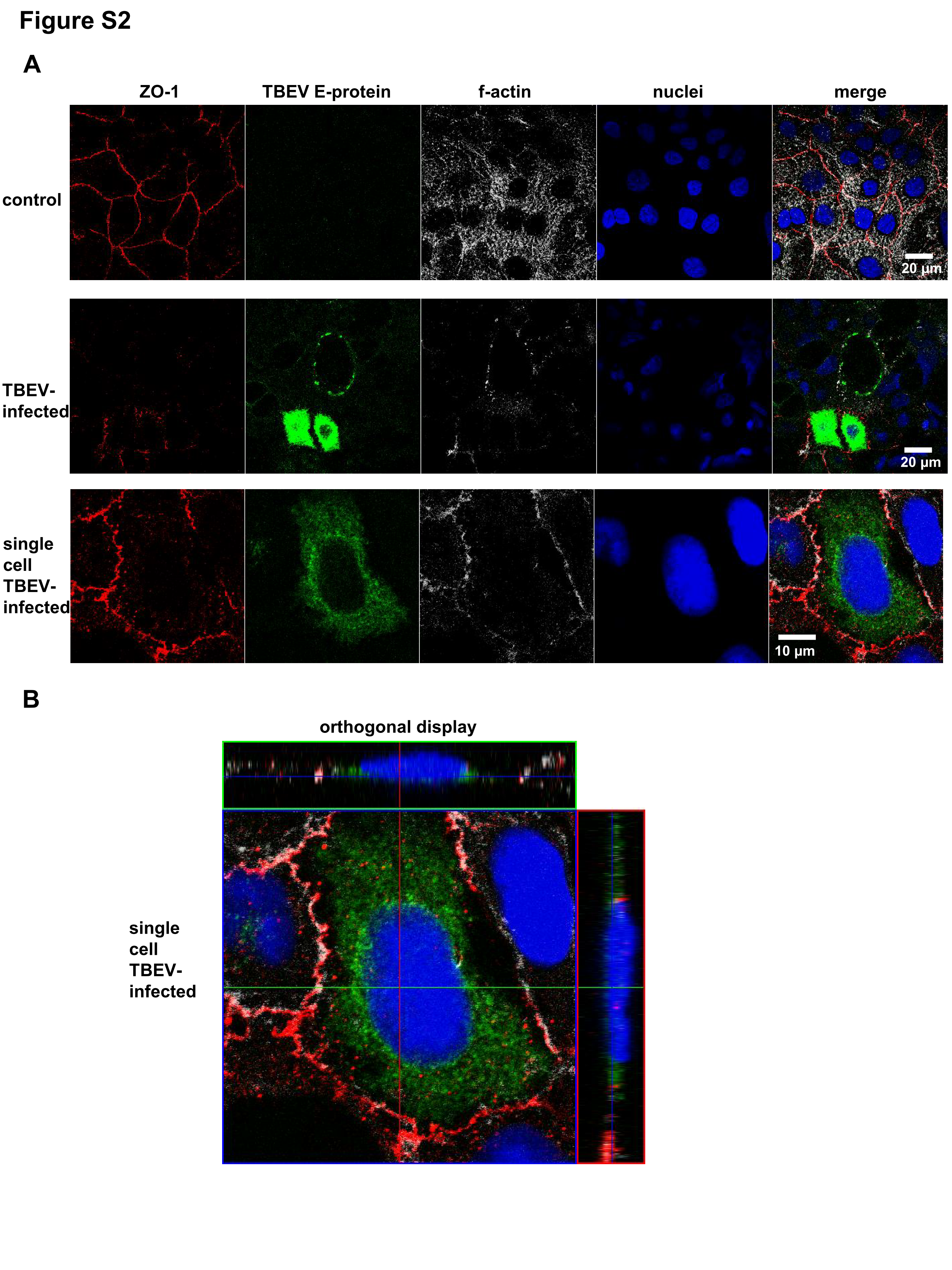

Supplement: Figure S2 — The effects of TBEV on tight junction changes may also contribute the drop in TER. Representative tight junction protein ZO-1 distribution and F-actin as cytoskeletal marker were stained in TBEV-infected and non-infected Caco-2 cells to display structural correlates to the electrophysiological findings. (A) ZO-1 and F-actin were disrupted by TBEV infection. Cells were fixed and stained for ZO-1 with primary antibodies and secondary anti-Rabbit Alexa Fluor 594 (red), TBEV E monoclonal antibody and anti- mouse conjugated with FITC (green). F-actin (white) stained with Atto-Phalloidin 647N (Sigma-Aldrich). Nuclei stained with DAPI (blue). Micrographs were taken by confocal microscopy. (B) Corresponding image of (A) as Z-stack in XY-plane. (TIF) [file pone.0096957.s002.tif]

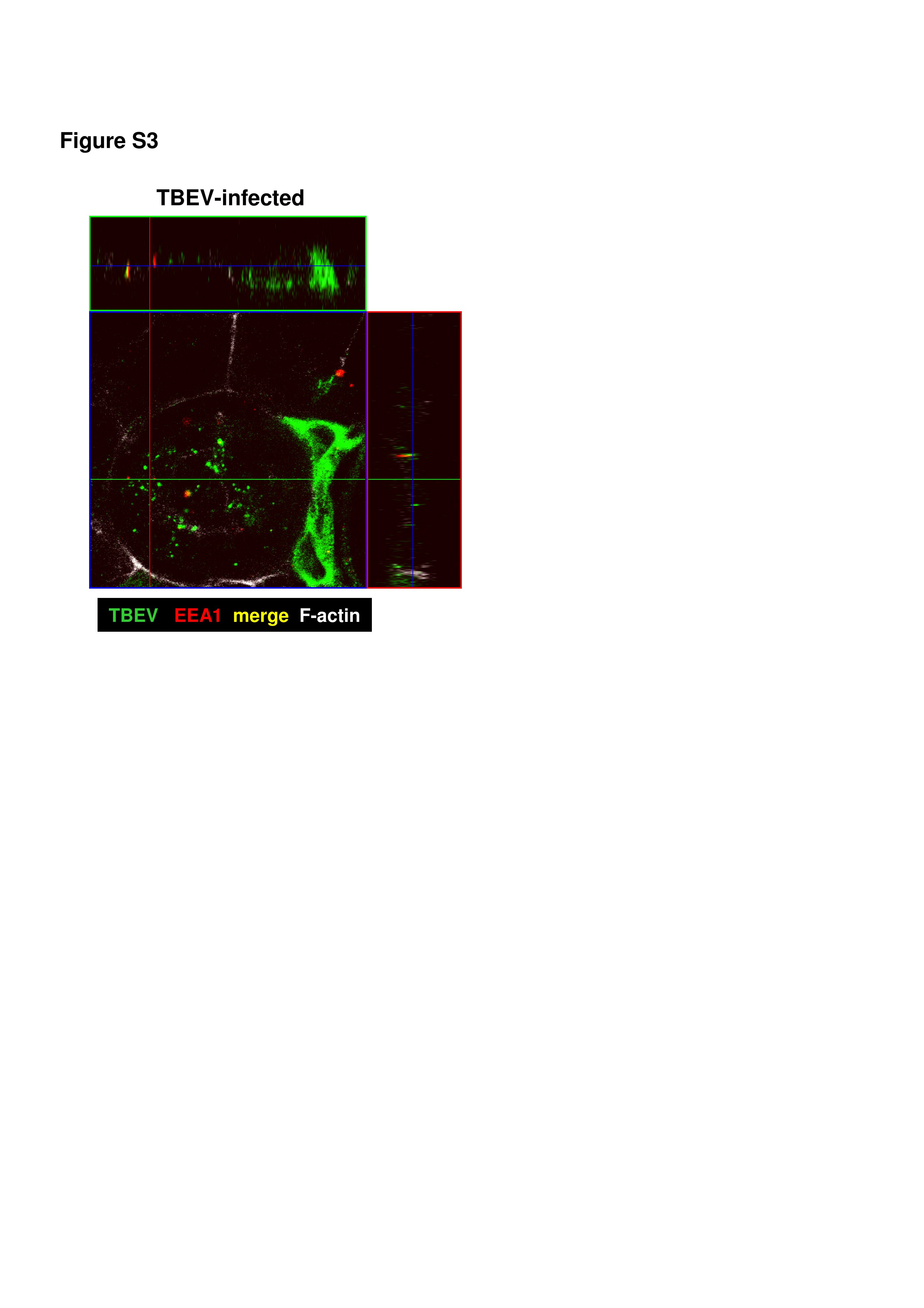

Supplement: Figure S3 — Co-localization of TBEV and EEA1. The infected cells were observed using confocal microscopy. The Z-stack image shows the virus co-localizes with EEA1 as yellow dots in XY-plane. (TIF) [file pone.0096957.s003.tif]

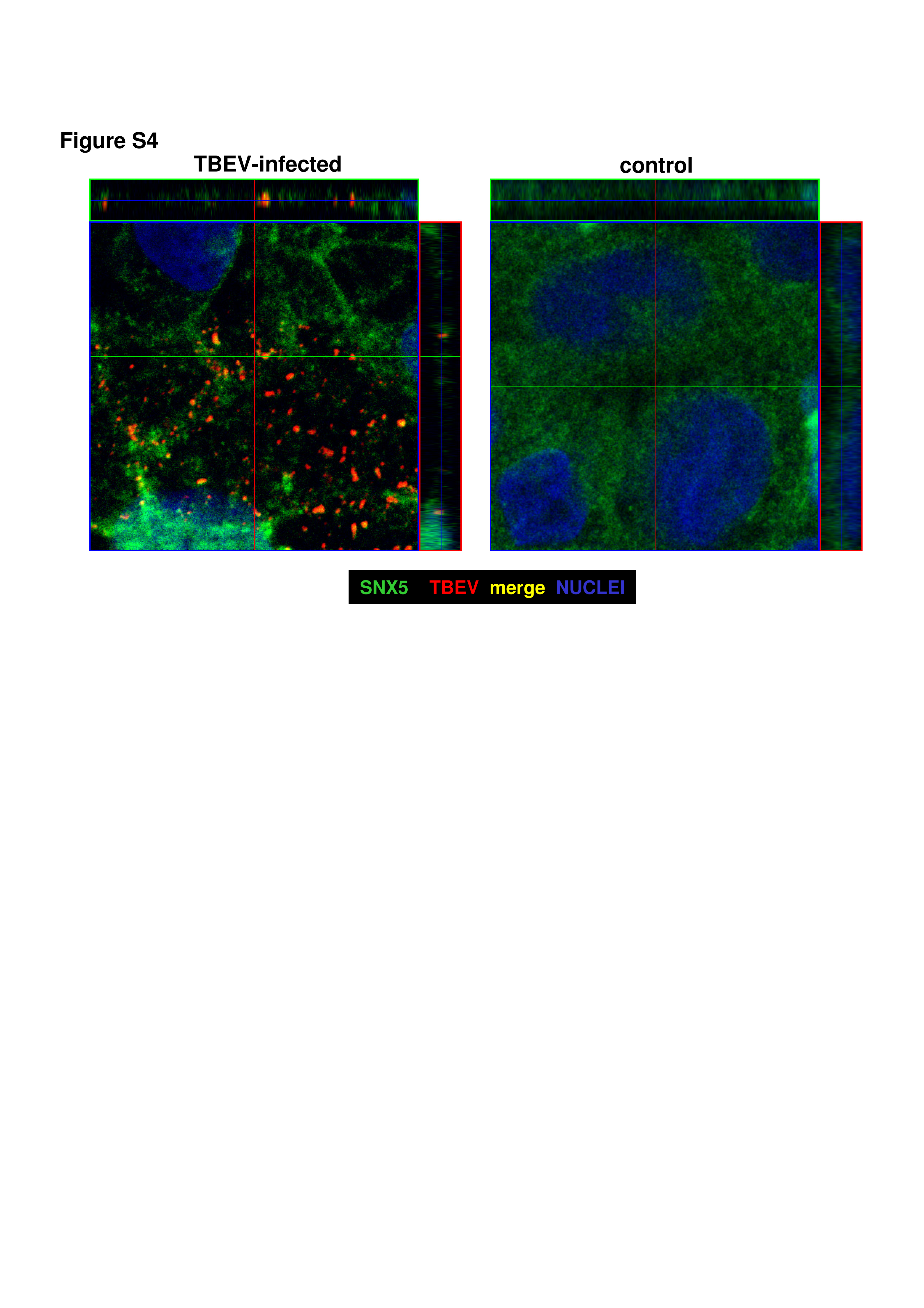

Supplement: Figure S4 — Co-localization of TBEV and SNX5. The Z-stack images in XY-plane were taken using confocal microscopy. The left image shows the virus co-localizes with SNX5 as yellow dots. In the right control image SNX5 is evenly distributed. (TIF) [file pone.0096957.s004.tif]
